# Novel fNIRS study on homogeneous symmetric feature-based transfer learning for brain–computer interface

**DOI:** 10.1038/s41598-022-06805-4

**Published:** 2022-02-24

**Authors:** Khurram Khalil, Umer Asgher, Yasar Ayaz

**Affiliations:** 1 National Center of Artificial Intelligence (NCAI), School of Mechanical and Manufacturing Engineering (SMME), National University of Sciences and Technology (NUST), Islamabad, 44000 Pakistan; 2 Department of Mechatronics Engineering, College of Electrical and Mechanical Engineering, National University of Sciences and Technology (NUST), Islamabad, 44000 Pakistan

**Keywords:** Brain-machine interface, Brain imaging

## Abstract

The brain–computer interface (BCI) provides an alternate means of communication between the brain and external devices by recognizing the brain activities and translating them into external commands. The functional Near-Infrared Spectroscopy (fNIRS) is becoming popular as a non-invasive modality for brain activity detection. The recent trends show that deep learning has significantly enhanced the performance of the BCI systems. But the inherent bottleneck for deep learning (in the domain of BCI) is the requirement of the vast amount of training data, lengthy recalibrating time, and expensive computational resources for training deep networks. Building a high-quality, large-scale annotated dataset for deep learning-based BCI systems is exceptionally tedious, complex, and expensive. This study investigates the novel application of transfer learning for fNIRS-based BCI to solve three objective functions (concerns), i.e., the problem of insufficient training data, reduced training time, and increased accuracy. We applied symmetric homogeneous feature-based transfer learning on convolutional neural network (CNN) designed explicitly for fNIRS data collected from twenty-six (26) participants performing the n-back task. The results suggested that the proposed method achieves the maximum saturated accuracy sooner and outperformed the traditional CNN model on averaged accuracy by 25.58% in the exact duration of training time, reducing the training time, recalibrating time, and computational resources.

## Introduction

Brain–computer interface (BCI) offers an interaction between the brain and external devices through signals generated from the brain without the peripheral nervous system’s involvement^1^. BCI is among such neurofeedback methods that may enhance patients’ quality of life suffering from acute motor debilities due to tetraplegia, stroke, and other spinal cord injuries^2^. More BCI applications are in areas of neuro-rehabilitation, communication and control, motor therapy and recovery, brain monitoring, and neuro-ergonomics^3–5^. Non-invasive neuroimaging modalities like functional magnetic resonance imaging (fMRI), electroencephalography (EEG), magnetoencephalography (MEG), and functional near-infrared spectroscopy (fNIRS) are greatly used in BCI systems for brain imaging and functional assessment of activities. Portable non-invasive neuroimaging techniques are generally preferred owing to their ease of use with fewer imaging protocols. The commonly used neuroimaging methodologies in this context are EEG and fNIRS. Both the modalities are portable and lightweight and require a small setup than the other techniques^6^. The electrodes capture EEG signals due to current neurons’ current variation due to postsynaptic activities^7^. While fNIRS constructs the brain’s functional neuroimages using near-infrared (NIR) light and gauge hemodynamic response function (HRF) in form of change in concentration of oxy and deoxygenated hemoglobin (HbO and HbR) to estimate the brain activities. Just like fMRI, the fNIRS also measures the blood oxygen level dependence (BOLD).

Using the BCI systems out of the laboratory needs to address several challenges such as robust signal acquisition, extracting required information from raw brain signals, and accurate control or command generation through data classification^8,9^. Another challenge hindering the BCI systems is the need for lengthy recalibration due to the high dimensionality and low signal-to-noise ratio (SNR) of EEG and fNIRS signals^10^. Typically, each new session’s calibration time for these modalities-based BCI systems takes up to 20–30 min approximately^11,12^. That extended time exhausts the subjects and puts extra fatigue even before the actual experimentation starts or even before the BCI system became fully functional. Another important factor is the non-stationary nature of brain signals. The exact brain state depends on mental and psychosomatic conditions, concentration level, factors like drowsiness, fatigue, anatomical differences, and statistical variations in the data^13,14^. The artifacts like instrumental noise, motion artifacts and poor sensitivity in naturalistic and non-structured environments, and the experimental errors due to variations in the electrodes’ resistivity may also alter the acquired brain signals^15–18^. All these factors result in a complex classification problem. To successfully classify the correct brain states, brain signals classification and neurofeedback are implemented in four stages: first is pre-processing, then feature extraction, classification, and lastly command generation^19,20^. The extracted features from brain signals are used to train the classifier. Machine or deep learning (DL) classifiers are used to discriminate various states of brain data. The different studies tried to address these challenges by exploiting various methods and algorithms while maintaining accuracy and information transfer rate (ITR) in a significant range^7,12,21–23^. Deep learning (DL) algorithms have been vigorously applied in different BCI studies such as an artificial neural network (ANN)^24,25^, convolutional neural networks (CNN)^26,27^, deep belief network (DBN)^28^, long short-term memory (LSTM)^29,30^, and cascade CNN-LSTM^31^. Although DL algorithms have superior learning capabilities and can address complex classification problems, at the same time, these algorithms have posed a unique challenge of Big Data in the BCI domain^19^. DL’s inherent bottleneck is the requirement of the huge amount of training data and computational resources for training deep networks^29^. The collection of a very large amount of neuroimaging data is very complicated and expensive in terms of time and resources, making it very hard to develop a substantial-scale, high-quality marked dataset for DL models’ training. Moreover, it is difficult to approximate probability distributions of the feature vectors from low SNR signals, mostly in the case of machine learning (ML) algorithms, where only a few trials are performed for multi-dimensional brain signals. All these factors lead to the poor performance of trained classifiers on new session data. In this scenario “Transfer learning” proved to be an encouraging approach candidate to deal with these problems.

Transfer learning algorithms used in EEG-based BCI are primarily based on two approaches, one is importance sampling cross-validation methods^20,32^, and the second is instance selection methods^33,34^. Covariate shift adaptation (CSA) proposed in the study^20^ uses the importance sampling cross-validation to weigh the data from the target domain (other subjects). The final prediction function is estimated based on parts with high weights, and others are rejected. In various studies^33,34^, trials are selected on an active learning base and based on an instance selection approach close to the new subject’s few informative trials. Then, to train the BCI model, selected trials based on an instance selection approach are added to the new subject’s existing labeled trials. In^35^, Zhang et al. proposed a diagnosis to susceptibility to alcoholism was done via extracting features using deep learning algorithms combined with transfer learning. Most of the proposed transfer learning algorithms in the feature domain focus on improving common spatial patterns (CSP). CSPs are improved with modifications of either the covariance matrix using the estimation method^36,37^, or the CSP optimization function^7,38^. An extension of CSP, proposed by Samek et al. in^39^, transferred stationary information instead of discriminative information across multiple subjects by learning a stationary subspace. Similarly, to solve the Motor imagery (MI) for BCI, the authors in^40^ proposed a combination of Continuous Wavelet Transform (CWT) along with deep learning-based transfer learning. Many existing MI-based BCI transfer learning algorithms on the classification domain have used domain adaptation techniques^41–43^ and ensemble learning of classifiers^7,23^. Domain adaptation techniques use the source domain classifier for the target domain while adjusting its parameters according to target data. Moreover, multi-task learning is also used in BCI^21,44^, where the classification parameters are learned together from multiple subjects, resulting in minimization of the average total errors and differences among the parameters of the separate classifiers. This approach was a success to some extent. Still, it had its constraints as many parameters needed to be optimized simultaneously, making it computationally expensive. Similarly, that approach does not consider the similarities and dissimilarities between the data within subjects during the learning process. Several studies on BCI for the classification of different controlled and uncontrolled cognitive tasks^29,45–50^ have used fNIRS. Despite being getting popular, to the best of the authors’ knowledge, there is no study on applying transfer learning in fNIRS-based BCI. The application of symmetric homogeneous feature-based transfer learning in the fNIRS domain is novel. This study’s major takeaway is that optimization obtained through transfer learning is superior to traditional DL network training.

Symmetric feature-based transfer learning approach discovers underlying meaningful structures between the domains to find a common latent feature space that has predictive qualities while reducing the marginal distribution between the domains^51^. The exchange learning approach proposed by Prettenhofer addresses the complicated situation of a source space containing marked and unlabeled information and a physical space containing unlabeled information. The auxiliary correspondence learning procedure from Blitzer is applied to this issue. Supplemental correspondence learning depends on the manual meaning of turn works that catch correspondence between the source and target spaces. Viable rotate capacities should utilize highlights that happen as often as possible in the two areas and have significant prescient characteristics. Each turn work is transformed into a linear classifier using information from the source and target spaces. From these turn classifiers, correspondences between highlights are found, and an inactive component space is found out. The unused component space is utilized to prepare the last objective classifier. The paper by Prettenhofer uses this answer to take care of the issue of text order where the source is written in one language, and the objective is written in an alternate style. In this particular execution alluded to as cross-language essential correspondence learning (CLSCL), the rotate capacities are characterized by sets of words, one from the objective and one from the source, that speaks to coordinate word interpretations from one language to the next. The tests are performed on the utilization of report assumption characterization and archive point arrangement. English archives are utilized in the source, and other language reports are being used in the objective. The benchmark technique used in this test prepares a student on the marked source records; at that point deciphers the objective reports to the source language and tests the translated form. An upper bound technique is set up via preparing a student with the named target archives furthermore, experimenting with the objective reports. Standard order precision is estimated as the exhibition metric^52^.

## Methods

### Dataset and data acquisition

**Figure 1:**
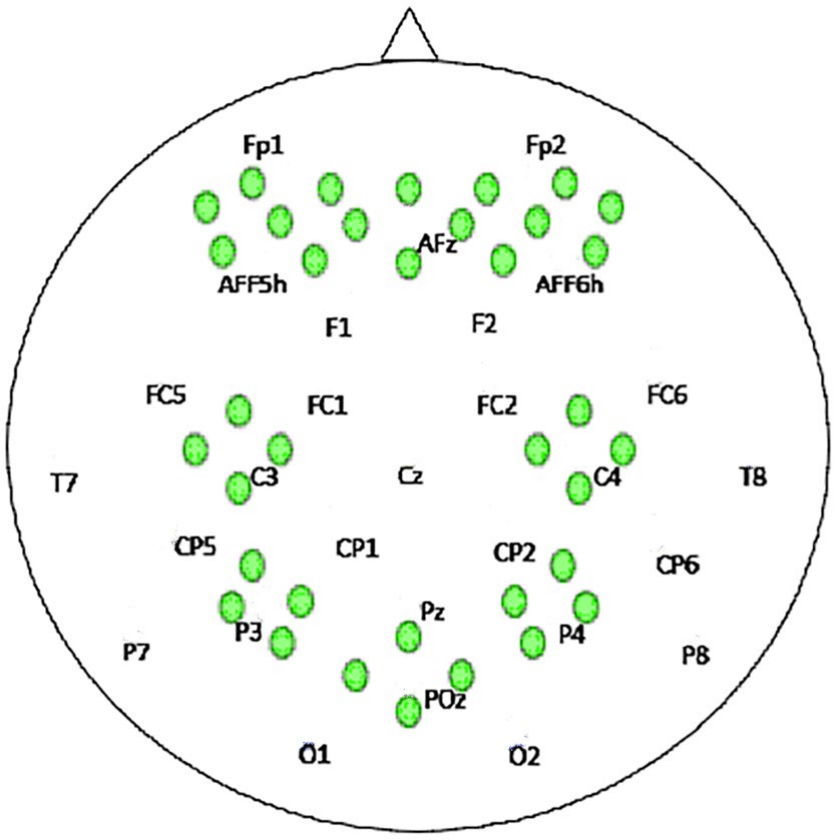
The NIRS optodes position according to 10-5 system.

This study used an open-source meta-dataset acquired at the Technische Universität Berlin by Jaeyoung Shin et al. in 2017^39,53^. The dataset includes fNIRS data of the scalp for different levels of mental workload acquired from 26 subjects. NIRScout (NIRx Medizintechnik GmbH, Berlin, Germany) was used for NIRS data acquisition using the configuration of 36 channels, according to the internationally recognized 10-5 system as shown in Fig. 1, at a sampling rate of 10.4 Hz. Sixteen optodes, a combination of sources with detectors were positioned at the frontal lobe across the region of AFz to AF8, and four channels were paced at C3, C4 for the motor cortex region. Four channels were places in the parietal region across P3 and P4. Likewise, four channels around the POz region for the occipital region. The distance between the source and the detectors was ensured to be 30 mm^40^. All participants were informed about the experimental procedure and gave written informed consent prior to the experiment. All the experiments were conducted in accordance with the Declaration of Helsinki and was approved by the Ethics Committee of the Institute of Psychology and Ergonomics, Berlin Institute of Technology.

### Experimental paradigm

**Figure 2:**
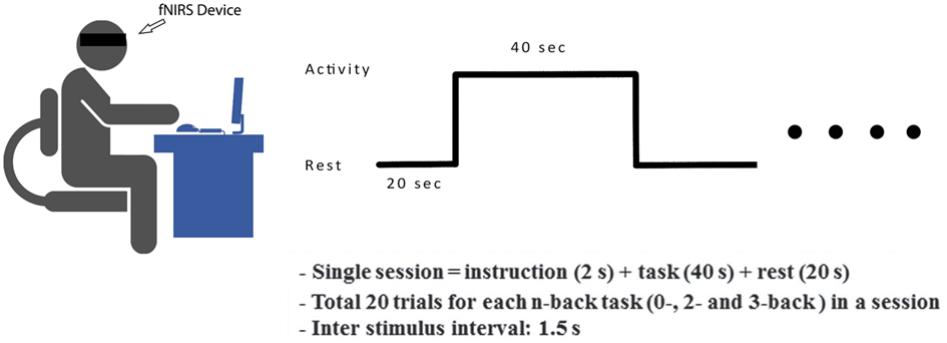
The experimental paradigm for data acquisition.

Before the experimentation starts, all subjects were seated in a comfortable chair in front of a 24-in. LCD monitor, placed at a distance of 120 cm. It is pertinent to mention that the distance between the subject and the monitor is 120 cm. Subjects were asked to press numeric keys 7 and 8 to record their response and to ensure the subject’s engagement during data acquisition, with their index and middle finger, using a keypad attached to their right side. Furthermore, subjects were instructed to stay focused during the experiment by restricting their eye movement only to the monitor in order to avoid motion artifacts. The experiment protocol was designed to perform three cognitive tasks i.e., n-back, Discrimination Selection Response (DSR), and Word Generation (W.G) by each subject. The tasks were performed in descending order depending on task difficulty level as due to the long duration of tasks and data recording, the subject’s focus decreases with apparent stress and fatigue. First task A was completed, then C, and lastly B. In this study, only dataset A (n-back) is used and is explained in detail in the next section. So, first, the n-back task was performed followed by W.G and lastly, data acquisition was done for DSR. For further information on other datasets (DSR and W.G) and analysis^39^. The time sequence of the designed n-back experiment is shown in Fig. 2.

The n-back dataset consists of three sessions where each session is comprising of three individual series for individual n-back tasks i.e., 0-back, 2-back, and 3-back tasks. So, for each subject total of nine n-back series (3 sessions × 3 series) were performed where a single series recording time was 62 s. In a single series, n-back task instructions were played for the first 2 s, followed by 20 trials of the n-back task for the next 40 s, and then the last 20 s were reserved for rest. In order to make the experiment more engaging and to keep the subject focused, a 250 ms beep was provided at the start and end of the task. Additionally, the word ‘STOP’ was also displayed at the end of the task followed by a fixation cross in the rest period to keep the subject focused and avoid unnecessary head and eye movements, and allowing the brain to relax to the standard baseline state. During the task period, subjects were asked to press either the target button using the right index finger or the non-target button using the right middle finger to record their response using the numeric keypad. In the case of the 0-back task, subjects were instructed to press the target button only in case if the number being displayed matched the last displayed number. Similarly, for 2 and 3 back tasks if the number being displayed matches the last 2 and 3 numbers displayed, respectively. The probability of appearing target vs. non-target numbers was 30%. The 0-back task was followed by the 2 and 3 back tasks. During these tasks, the subjects were asked to press the target button only in the case that if the number being displayed matches the 2 and 3 last numbers. The fixation cross followed the task period; the subjects were instructed to gaze at the cross and relax. It allowed the brain state to return to the standard baseline value^53^. As there were three sessions, each having three series, while every single series encompassed 20 trials, making 180 trials.

### Data pre-processing

The acquired data were first translated to the oxy and deoxy-hemoglobin (HbO and HbR) intensity variations to pre-process the fNIRS data. The conversions were made through the modified Beer-Lambert law (MBLL)^41^. The fNIRS raw data were acquired and sampled at 10 Hz. This dataset’s fundamental frequency was very low, so the down sampled was not fed into the Butterworth bandpass filter. Instead, low pass filtered is employed to avoid losing the essential frequency component^42^. The cutoff frequency of the filter was set to 0.2 Hz to remove the artifacts due to blood pressure, heartbeat and breathing and high frequency instruments.

### Proposed convolutional neural network model

In this study, a convolutional neural network (CNN) was used to classify three mental workloads (MWL) classes owing to its reputation and increase in use for different MWL classification studies^26,27,29^. CNN is a deep neural network that may integrate one or more convolutional layers with a pooling layer, batch norm layer, activation layer, dense layer, and at very last an output layer. The most important CNN layer, i.e., the convolutional layer, allows its inputs to pass through cascaded filters bank and performs simple convolution operations. Essentially convolution layers output feature maps extracted from the input due to convolution, i.e., shifting and multiplication of input signal and filter^43^. These feature maps are then used as an input to the next layer in the CNN architecture or as a set of definitive key features on which classification is performed in the last fully connected layers.

1 $$Output \; size\, \left(W,H\right)= \frac{(N-F)}{Stride }+1$$

where *W* and *H* are the width and height of the output activation map or feature map, *N* is the dimension of the input activation or feature map, *F* is the dimension of filter sliding over the input image or activation map, the stride is the number of steps taken while sliding filter. While the parameters of a layer are calculated using:

2 $$Parameters=\left(W*H*K\right)+K \,biases$$

W and H are the width and height of the output activation map or feature map, K is the number of filters, and K biases are the number of biases.

**Figure 3:**
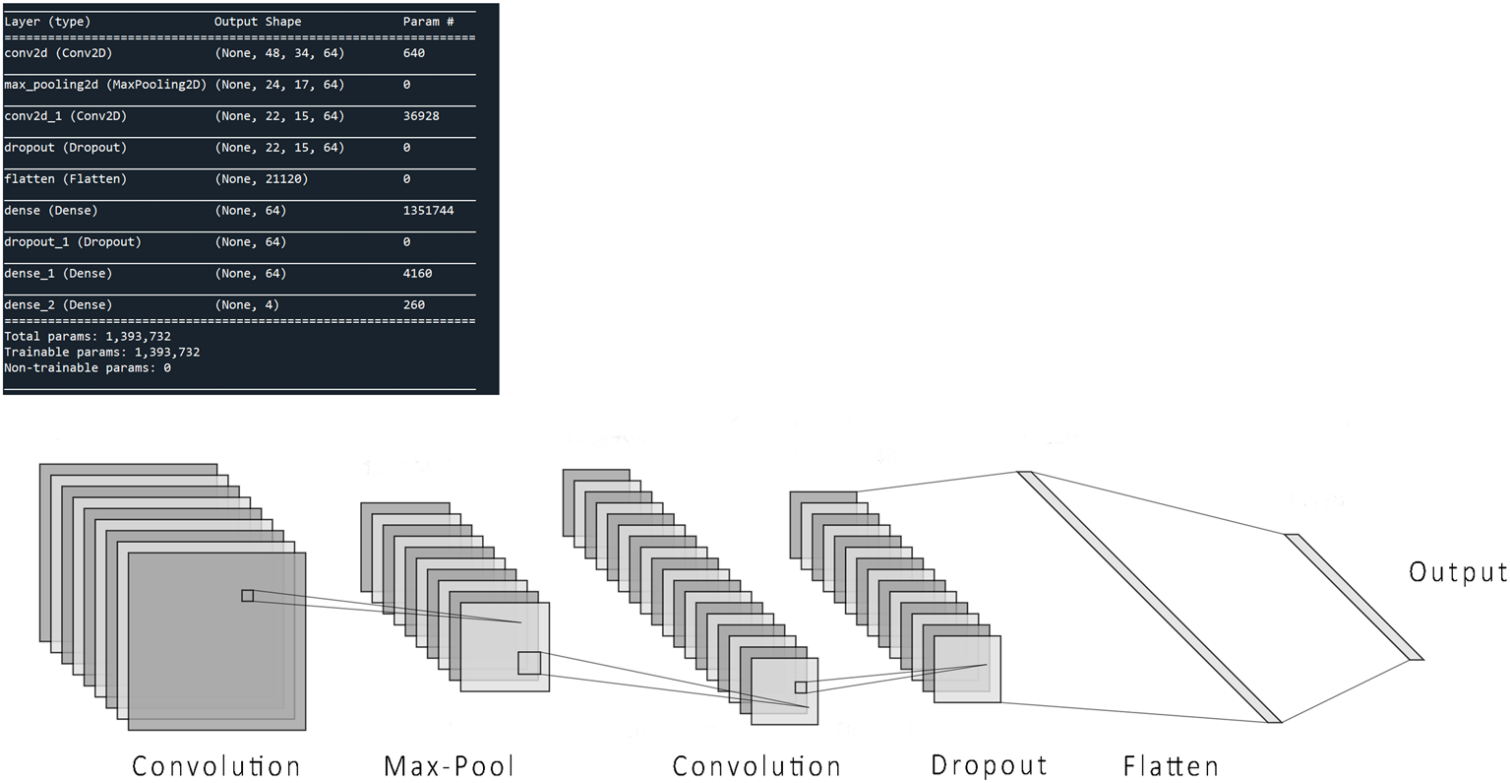
The proposed CNN model with input, convolution, max pool, dense, and output layers. Model summary includes details about hyperparameters and network architecture.

The mathematical formulation of CNN layers is well explained in^44^. During the CNN model training, both filter bank parameters and dense layer weights are adjusted throughout the period. The model precisely fits the training dataset with the least possible error. Successful implementation of CNN for a given dataset mainly relies on the fact that different data domains usually have some standard key features shared across all of its elements (such as images). But this is not the case when it comes to generalization in areas with high inter-subject unpredictability like brain signals acquired with EEG, fNIRS, fMRI methodologies, where data differ from subject to subject and depend on a lot of external and internal factors^45^. The research’s CNN models are based on a feed-forward CNN architecture comprising pairs of convolution and pooling layers^46^. After initial tests on different feed-forward CNN architectures, the chosen CNN architecture with complete parameters and structure is shown in Fig. 3. A fully connected feed-forward CNN network is selected with two convolution layers, a max-pooling layer, followed by a flattening and dense layer. Finally, a fully connected layer ends into the final output layer.

### Transfer learning

Since the study is based on homogeneous transfer learning, it is assumed that multiple fNIRS sessions previously acquired from different subjects or the same subject on the same or different tasks are already acquired. Throughout the literature, various research studies used different terminologies for similar concepts of transfer learning, types of transfer learning, and their mathematical formulation like domain adaptation, knowledge transfer, and transfer learning; following definition of transfer learning is used in this study:

A domain D consists of two essential parts, a feature space also known as latent space X and a marginal probability distribution (MPD) *P(X),* where feature vectors *X* = *{*× *1, . . . , xn} ∈ X*. In the case of BCI, the generation of command is the classification goal, and the channel readings are considered as features, then *x*_*i*_ is the *ith* feature vector (instance) corresponding to the ith generated command, *n* is the numbers of feature vectors in *X*, and the ***X*** is the space of all possible feature vectors. For a given domain *D*, a task *T* can be defined as a label space *Y* and a predictive function *F* < *.* > *.* The predictive function *F* < *.* > is learned from the feature instance and corresponding label pairs *[x*_*i*_*, y*_*i*_*]* where *x*_*i*_* ∈ X* and *y*_*i*_* ∈ Y*. In the BCI problem, *Y* is the set of labels that might be *rest, open, close* commands, *y*_*i*_ takes on one of the command values, and *f(x)* is the function approximator that predicts the label value for the command classification *x*. From the above definitions, a data domain is given by *D* = *[X, P(X)]*, and a task is provided by *T* = *[Y, F* < *.* >*].* Also, for consistency, we will represent source domain data as *D.S.*, and by definition, it will be given by *D.S.* = *[(x*_*S1*_*, y*_*S1*_*). . . , (x*_*Sn*_*, y*_*Sn*_*)],* where *x*_*Si*_* ∈ X.S.* and it is the ith data point of *D.S.* and *y*_*Si*_* ∈ Y.S*. is the corresponding feature label for *x*_*Si*_. Likewise, the target domain data can be given as *D.T.* where *D.T.* = *[(x*_*T1*_*, y*_*T1*_*). . . , (x*_*Tn*_*, y*_*Tn*_*)]* where *x*_*Ti*_* ∈ X.T*. and it is the *ith* data point of *D.T.* and *y*_*Ti*_*, ∈ Y.T*. is the corresponding class label for *x*_*Ti*_. Now, the source task, the target task, the source predictive function, and the target predictive function can be represented by *T.S., T.T., F.S.* < *.* > *,* and *F.T.* < *.* > , respectively. Now we can define transfer learning as improving the *F.T.* < *.* > , target predictive function, by using the gathered information from source domain data *D.S.* and source task *T.S.*, given source domain *D.S* and target domain *D.T.* with or without target tasks *T.T*. Transfer learning can be categorized into two types: (1) Homogenous transfer learning and (2) Heterogeneous transfer learning. Mathematically, the condition where the source and target domain features *Xt* and *Xs* are equal for transfer learning is called homogenous transfer learning^47^. Whereas the state where the source and target domain features *Xt* and *Xs* are not similar is called heterogeneous transfer learning. Homogenous and heterogeneous transfer learning is also called intra-domain and inter-domain transfer learning, respectively. This study performed homogenous transfer learning on fNIRS data and evaluated its performance and viability for deep learning networks.

## Methodology

The available dataset of 26 participants is divided into three subsets with an approximately 60:20:20 ratio. The first 16 participants’ data is used to train the CNN network to learn the task’s domain knowledge. This trained network is then used as parameters trained on *D.S*. transferred to *D.T.* The validity and viability of transfer learning are evaluated under the following assumption: the transfer learning efficiently transferred the source domain knowledge to the target domain, it required the reduced training iterations for deep learning models, and while transferring the learned domain knowledge, the transfer learning increases the achieved classification accuracies. We evaluated these assumptions by placing the remaining ten subject data into two groups and named them as the baseline and control groups. The baseline group is used for training conventional deep neural network models in a standard and widely adapted setting. The aim of this study is to learn the intra-subject varainces while performing the same task, as evident in the name homogenous transfer learning. We intended to learn features that maximally differentiate the n-back classes (0,2,3-back and rest) for the new subject in least amount of training data and time. For the training of the trained CNN network, we performed experiments with 70:30 split ratio, leave one out (LOO) and tenfold cross-validation methods. The result obtainerd with tenfold cross validation results were the best performing one. In contrast, the control group is retrained and fine-tuned on the pre-trained CNN model with domain knowledge Ds and Ts from the first 16 participants. The pre-trained model is fed with the control group data *D.T.* and trained with different epochs from 10 up to 60. The different experiments were performed for the retraining and fine-tuning process. First of all, the complete learnable parameters of the trained CNN were freezed except the last dense layer, and retraining was performed. Next, we repeated the same experiment by unfreezing the last two dense layers. The process is repeated up till the first convolutional layer. The retraining by unfreezing the last two dense layers yielded the best results and is used in further analysis. These obtained accuracies are compared with the baseline group accuracies. The statistical analysis is performed on the obtained accuracies, and the conclusion is discussed in the next section.

## Results

### Statistical analysis

**Figure 4:**
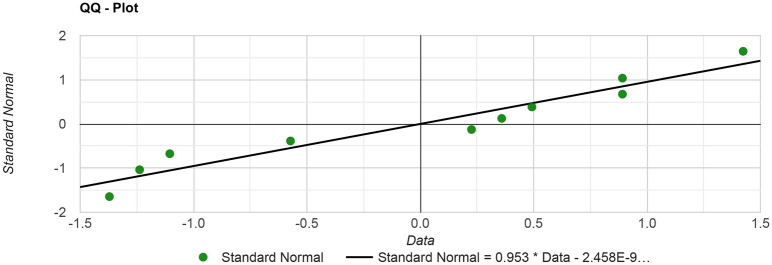
The depiction of accuracies normality using QQ plot.

The statistical analysis was performed between baseline and control groups (reference). Shapiro–Wilk test is used to gauge the normal distribution of baseline and control groups’ accuracies. It is a right-tailed normal distribution criterion with a null and alternate hypothesis as the H_0_ (null hypothesis): if the population is normally distributed and H_1_: if the population is not normally distributed^48^. For all epochs, the resulting p-value is (probability with data normal distribution with the confidence of 95%) > *α* (confidence level); therefore, the H_0_ is accepted. The quantile–quantile or QQ-plot is another method used for a graphical illustration of the Shapiro–Wilk test and shows the significance test run on the baseline group as shown in Fig. 4. Based on the Shapiro Wilk test results, it became established that the statistical significance measures with other scales like t-test and ANOVA are possible on the current accuracies. The paired t-test is calculated with the null hypothesis (H_0_): There is no significant difference between the baseline and control group accuracies, and the p-value > 0.05 and the alternative hypothesis (H_1_): the two populations are not equal, a significant difference between these accuracies and the p-value < 0.05. The two-tailed P-value (2.443e−8) is less than 0.0001 with the degree of freedom (DOF) = 9. By conventional criteria, this difference is considered to be extremely statistically significant. After analysis, the t-value comes out to be t = 17.8723 . The null hypothesis (H_0_) is rejected with p < 0.05, and the alternate hypothesis is accepted.

## Findings

**Figure 5:**
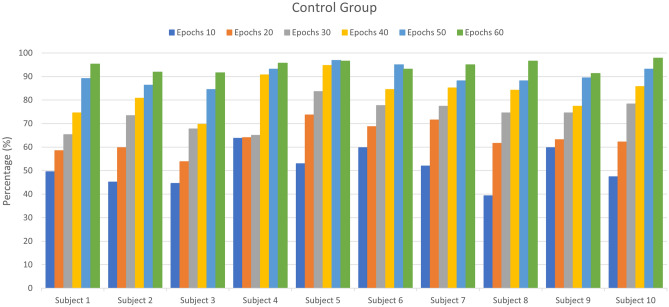
The accuracies obtained by the learned CNN model on control group subjects at epochs from 10 up to 60.

**Figure 6:**
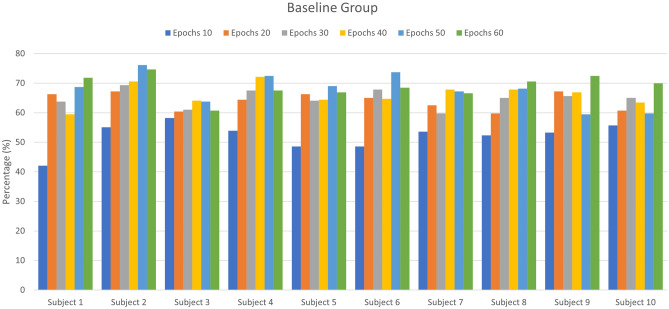
The accuracies using CNN model on baseline group subjects at epochs from 10 up to 60.

**Figure 7:**
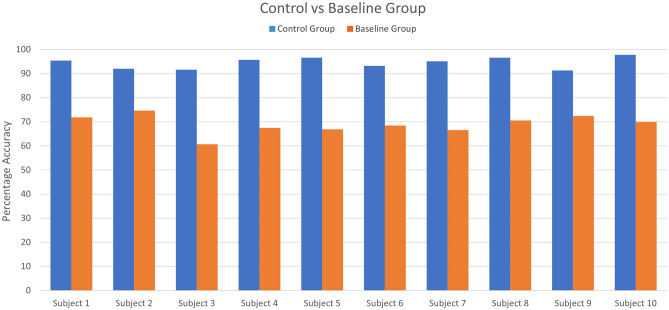
The comparison between accuracies acquired on control and baseline group via learned CNN and randomly initialized CNN network.

This section presents the results of transfer learning for fNIRS-based BCI after experimentation and statistical analysis. The first 16 participants’ data is used to train the CNN network to learn the task’s domain knowledge. This pre-trained CNN model is used to re-train and fine-tune the learned parameters on the control group. The CNN network is trained and tested on both baseline and control groups with tenfold cross-validation. Figure 5 shows the accuracy of control group subjects with different training epochs (n = 10, 20, 30… 60), while Fig. 6 shows the accuracy of baseline group subjects trained on the randomly initialized CNN network. Tables 1 and 2 represent the accuracies of the control group and the baseline group, respectively. Both networks are trained with a range of epochs from 10 to 60 with an increment of 10 epochs per step. The optimality of network models is measured by the ‘accuracy’ metric that tells the percentage of true positives from all predictions. The average accuracies for the control group after each 10 step epochs were 51.42, 63.72, 73.78, 82.76, 90.43, and 94. %, while for the baseline group, the average accuracies were 52.14, 63.96, 64.89, 66.13, 67.83, and 68.95%. After 60 epochs, the training is stopped because the pre-trained CNN starts over-fitting. The results show that the proposed technique successfully transferred the learned knowledge and achieved the maximum accuracy of 97.83%. The control group’s saturated accuracy results are obtained earlier than the conventional CNN on the baseline group, which significantly reduces the number of training epochs and effectively reduces the time required to train the network. The proposed transfer learning method also outperformed the averaged accuracy achieved using the learned CNN model over the traditional CNN model by 25.58% in the exact duration of training time as shown in Fig. 7. The Keras is used for prototyping with the TensorFlow backend. The networks are trained on Nvidia GEFORCE GTX 1060 GPU, having 3 GB VRAM on spyder IDE. The number of neurons, the number of filters, the number of layers, their combinations, dropout, and max-pooling percentage, etc., all remain to be at best ‘hyper parameters’. For this study, the network architecture design process was as follows: create a network with a minimum number of parameters, a single convolutional layer, a single pooling layer, and one dense layer, then tune other hyperparameters. Add more layers and then tune network hyperparameters with grid search using the sklearn wrapper and choose the best performing network.

**Table 1:** The accuracies obtained on the control group from a range of epochs up to 60 (with increment of 10).

	Control group					
	Epoch 10	Epoch 20	Epoch 30	Epoch 40	Epoch 50	Epoch 60
Subject 1	49.54	58.51	65.33	74.61	89.16	95.36
Subject 2	45.20	59.75	73.37	80.80	86.38	91.95
Subject 3	44.58	53.87	67.80	69.66	84.52	91.64
Subject 4	63.78	64.09	65.02	90.71	93.19	95.67
Subject 5	52.94	73.68	83.59	94.74	96.90	96.59
Subject 6	59.75	68.73	77.71	84.52	95.05	93.19
Subject 7	52.01	71.52	77.40	85.14	88.24	95.05
Subject 8	39.32	61.61	74.61	84.21	88.24	96.59
Subject 9	59.75	63.16	74.61	77.40	89.47	91.33
Subject 10	47.37	62.23	78.33	85.76	93.19	97.83

**Table 2:** The accuracies with CNN on the baseline group from a range of epochs up to 60 (with an increment of 10).

	Baseline group					
	Epoch 10	Epoch 20	Epoch 30	Epoch 40	Epoch 50	Epoch 60
Subject 1	42.11	66.25	63.78	59.44	68.73	71.83
Subject 2	55.11	67.18	69.35	70.59	76.16	74.61
Subject 3	58.20	60.37	60.99	64.09	63.78	60.68
Subject 4	53.87	64.40	67.49	72.14	72.45	67.49
Subject 5	48.61	66.25	64.09	64.40	69.04	66.87
Subject 6	48.61	65.02	67.80	64.71	73.68	68.42
Subject 7	53.56	62.54	59.75	67.80	67.18	66.56
Subject 8	52.32	59.75	65.02	67.80	68.11	70.59
Subject 9	53.25	67.18	65.63	66.87	59.44	72.45
Subject 10	55.73	60.68	65.02	63.47	59.75	69.97

Machine or deep learning (DL) classifiers are used in various other studies to discriminate various states of brain data. The different studies tried to address these challenges by exploiting various methods and algorithms while maintaining accuracy and information transfer rate (ITR) in a significant range^7,12,21–23^. Deep learning (DL) algorithms have been vigorously applied in different BCI studies such as an artificial neural network (ANN)^24,25^, convolutional neural networks (CNN)^26,27^, deep belief network (DBN)^28^, long short-term memory (LSTM)^29,30^, and cascade CNN-LSTM^31^. Although DL algorithms have superior learning capabilities and can address complex classification problems, at the same time, these algorithms have posed a unique challenge of Big Data in the BCI domain^19^. DL’s inherent bottleneck is the requirement of a huge amount of training data and computational resources for training deep networks^29^. The collection of a very large amount of neuroimaging data is very complicated and expensive in terms of time and resources, making it very hard to develop a substantial-scale, high-quality marked dataset for DL models’ training. Moreover, it is difficult to approximate probability distributions of the feature vectors from low SNR signals, mostly in the case of machine learning (ML) algorithms, where only a few trials are performed for multi-dimensional brain signals. All these factors lead to the poor performance of trained classifiers on new session data. In this scenario “Transfer learning” proved to be an encouraging approach candidate to deal with these problems.

## Discussion

Transfer learning aims to produce an efficient model to map the learned knowledge from a source domain task to a different but related target domain task^49,50^. Training deep learning models only on target tasks may result in degraded performance due to insufficient data or labeled instances. Transfer learning improves the model’s ability to classify target instances by utilizing the source domain knowledge^54^. With the inherent constraints of collecting neuroimaging data and the high training iterations for deep learning models in BCI, transfer learning provides promising results. The collection of neuroimaging data is very complicated and expensive both in terms of time and resources, making it very hard to develop a substantial-scale, high-quality marked dataset for the training of deep learning models.

Usually while using machine learning algorithms on multi-dimensional brain signals, it is often difficult to approximate probability distributions of the features from low SNR signals with only a few trials. However, in this case the model’s ability to classify target instances can be improved by enhancing the training with supplementary labeled data from a related source domain. In last few years, various researchers have tested different transfer learning approaches on EEG-based BCI^20,32–34^. But the real challenge arises while distinguishing inherent cross-domain noise due to the varied distributions from the beneficial knowledge in a source domain and then applying that knowledge to a target domain. According to literature, transfer learning can be split into two main categories according to the feature space: homogeneous and heterogeneous transfer learning^55^. In homogeneous transfer learning, the feature spaces of the source and target domains is of the same dimension (Ds = Dt) while the data of both domains is represented by the same attributes (Xs = Xt) and labels (Ys = Yt). Thus, homogeneous transfer learning aims to bridge the gap in the data distributions experienced during cross-domain transfer^55^. While, in Heterogeneous Transfer Learning, the feature spaces between the source and target are non-equivalent and are non-overlapping i.e., Xs ≠ Xt and/or Ys ≠ Yt. The source and target domains may share no features or labels, and the feature spaces' dimensions also may differ. Thus, for cross-domain transfer, Heterogeneous Transfer Learning requires feature and label space transformations to bridge the gap for knowledge transfer and to handle the cross-domain data distribution differences.

For EEG-based BCI, both homogenous and heterogeneous transfer learning approaches are used in literature i.e., instance-based, feature-based, and parameter-based transfer learning^7,19,49^. Every transfer learning approach focuses on improving target prediction function using source and target domain data differently. Like, the instance-based transfer learning approach assumes that although the entire source domain cannot be used directly but some source domain data can be re-used for learning the target domain i.e., by combining the few target labeled data with some instances from the source domain, by some weight adjustments, if needed. While, the feature-representation transfer learning approach focuses on improving the construction of feature space for the target domain using the source domains’ data instead of combining target labeled data with source domain data to improve target prediction function^19^. The performance of the target task is thus enhanced by minimizing classification errors. Lastly, the parameter-based transfer learning relates target domain with ethe source domain by assuming that parameters and prior distributions are shared between the source’s functions and target tasks thus can be transferred to the target prediction function resulting in reduction of the classification errors.

In most of the transfer learning BCI approaches, some sort of knowledge is transferred between a source and target domain either by (i) finding some structure in the data that is invariant across datasets and known as stationary information transferred, (ii) finding some structure in a way the decision rules differ between different subjects and known as discriminative information transfer^37^. Here, the focus is on constructing discriminative systems by exploiting the features, filters, and classifiers to transfer stationary information. While in the case of discriminative information transfer, the aim is to construct more invariant systems that rely on common information across the source and target domains^56^. Due to the popularity of machine learning algorithms in BCI, various researchers opted for experimentation with transfer learning for the machine learning classifiers^7,20,33,36^. In^20^, authors proposed an instance-based transfer learning method, namely Bagged importance-weighted LDA (Bagged IWLDA), based on the covariant shift adaptation method. The purpose was to reduce the non-stationarities present in the recording of the different sessions. Another study^33^ proposed an instance-based transfer learning method based on active transfer learning (ATL) to transfer particular instances i.e., to find the most informative samples for labeling. This approach results a higher performance learning process with less labeling effort. In literature^7,36^ researchers have proposed different feature-based transfer learning methods for EEG-based BCI studies. Among all the presented methods in literature, spatial filters are most commonly used to learn the new feature representation for BCI transfer learning. Over the years different algorithms are designed to compute spatial features. While, Common Spatial Patterns (CSP), is the most commonly used algorithm of all for extracting discriminative features from EEG signals. Despite of its popularity among the researchers, the main bottleneck is its overfitting when there are only a few trials of data is available for training. Therefore, different improved approaches for CSP were proposed to overcome this limitation. In^7^ and^36^, linear discriminant analysis and stationary subspace-based CSPs were proposed, respectively. More precisely, it was proposed that using the data from a subset of source subjects could improve the CSP covariance matrix estimation. These studies showed that by using smart methods alongside CSP, this problem could be overcome.

This study proposed a novel symmetric homogeneous feature-based transfer learning methodology in the classification realm to increase the fNIRS-based BCI performance by reducing the training time, addressing the problem of insufficient data, and increasing the accuracy. The symmetric homogeneous feature-based transfer learning is applied in the following steps: A deep learning convolutional neural network (CNN) model is trained on multi-subject data acquired with the fNIRS system from subjects during Mental workload “n-back” tasks. Second, the trained CNN model parameters are transferred to train and fine-tune the unseen subjects’ data. Last, the transferred model’s learned feature space is utilized to regularize the re-training and fine-tuning process. The results confirmed that the proposed technique successfully transferred the learned knowledge and achieved the maximum accuracy of 96.5% with 20 epochs earlier than the conventional DNN method. The proposed transfer learning method also outperformed the averaged accuracy achieved using the learned CNN model over the traditional CNN model by 24.5% in the same duration of training time.

## Conclusion

In this research study, a feature-based homogenous transfer learning approach was explored for the classification domain to reduce the training and calibration time for the fNIRS-based BCI systems. We evaluated the validity and viability of transfer learning for the fNIRS-based BCI systems under the following different assumptions. First, the transfer learning efficiently transferred the source domain knowledge to the target domain and required reduced training iterations for deep learning models. Second, transfer learning minimizes the need for a large amount of data needed for training deep learning models for the target domain. We used 16 subjects to train the CNN network and named it a ‘learned CNN’ network that learns the source domain knowledge of the n-back dataset. Further, we split the remaining ten subjects into two groups, i.e., the control and baseline group. The control group is trained with the learned CNN network and baseline with a randomly initialized CNN network, and their accuracies are compared using statistical analysis. The results suggested that applying the proposed feature-based transfer learning algorithms could achieve the maximum saturated accuracy sooner than the baseline group, which reduces the training time. The proposed transfer learning method also outperformed the averaged accuracy achieved using the novel learned CNN model (94.52%) over the traditional CNN model (68.94%) by 25.58%. Thus, the proposed transfer learning methodology for fNIRS is a promising solution for both the problems of increased training iterations for deep learning models and limited training datasets for BCI.

The classification of different brain activities and training time of BCI models would remain an area of concern, leaving room for more research in using transfer learning methodology for fNIRS-based BCI. This study utilized the data acquired on the same task from different subjects. Future research work may explore the domains of the intrasession BCI dataset with heterogeneous transfer learning approaches. Additional experimentation of transfer learning for deep neural networks (DNN) designed explicitly for time-series data such as Long Short-Term Memory (LSTM) may be used to explore more optimal results with DNN compared to other machine learning classifiers. This study serves as a baseline study for future transfer learning research in fNIRS-based BCI.
